# Non-Dipping Blood Pressure Profile in Narcolepsy with Cataplexy

**DOI:** 10.1371/journal.pone.0038977

**Published:** 2012-06-29

**Authors:** Yves Dauvilliers, Isabelle Jaussent, Benjamin Krams, Sabine Scholz, Stéphane Lado, Patrick Levy, Jean Louis Pepin

**Affiliations:** 1 Sleep Unit, Department of Neurology, Hôpital-Gui-de Chauliac, CHU Montpellier, National Reference Network for Narcolepsy, Montpellier, France; 2 Inserm, U1061, Montpellier, France; 3 Univ Montpellier 1, Montpellier, France; 4 INSERM 1042, Hypoxia PathoPhysiology (HP2) Laboratory, Joseph Fourier University, Sleep Laboratory, Universitary Hospital of Grenoble, Grenoble, France; Tokyo Metropolitan Institute of Medical Science, Japan

## Abstract

**Background:**

Patients with narcolepsy-cataplexy (NC) mostly exhibit undetectable hypocretin levels. Hypocretin system is one of the key players in the complex interaction between sleep and the cardiovascular system. We tested the hypothesis that hypocretin deficiency affects cardiovascular risk factors by measuring nighttime and daytime ambulatory blood pressure (BP) and the night-to-day BP ratio as well as endothelial dysfunction by the digital pulse amplitude response in drug-free patients with NC compared to controls.

**Methodology:**

Sleep, clinical and biological cardiovascular risk factors, fingertip peripheral arterial tonometry, and 24-hour ambulatory BP monitoring were recorded in 50 drug-free patients with NC and 42 healthy control subjects, except for BP monitoring available in all controls but in 36 patients with NC.

**Principal Findings:**

More patients than controls were smokers, obese and with dyslipidemia. One-third of patients with NC were “non-dippers” (defined as <10% drop in BP during sleep) compared to only 3% of controls. The diastolic non-dipper BP profile had up to 12-fold higher odds of being associated with NC. We noted negative correlations between mean diastolic BP fall during night, REM sleep percentage and number of sleep onset REM periods, and a positive correlation with mean sleep latency on the MSLT. The digital pulse amplitude response measured by fingertip was similar between NC and controls.

**Conclusion:**

We found a high percentage of non-dippers in patients with NC with association with REM sleep dysregulation. The blunted sleep-related BP dip in NC may be of clinical relevance, as it may indicate increased risk for cardiovascular events.

## Introduction

Narcolepsy with cataplexy (NC) is characterized by excessive daytime sleepiness (EDS), cataplexy, and disturbed nocturnal sleep, including parasomnias, obstructive sleep apnea syndrome, and periodic leg movements [1]. Furthermore, frequent occurrences of rapid eye movement (REM) sleep onset periods during daytime and numerous awakenings during nocturnal sleep induce disruption of the sleep-wake cycle [1], [2].

Marked decreases in hypocretin-1 in the cerebrospinal fluid and in the number of hypocretin neurons have been demonstrated in NC [1],[3],[4]. The hypocretin neurons are located exclusively in the lateral hypothalamus, but project widely throughout the central nervous system, including hypothalamic and brainstem structures known to participate in central autonomic and cardiovascular regulation [5], [6].

Animal studies have explored the cardiovascular status of hypocretin-deficient rodent during wakefulness, showing lower arterial blood pressure (BP) compared to wild type [7], [8]. More recently, blunted NREM and REM sleep-related decreases in BP have been shown in hypocretin-deficient mice [9]. Pharmacological studies in mice or rats have revealed that the administration of hypocretin stimulates arousal and elevates arterial blood pressure, heart rate (HR), oxygen consumption, body temperature, and plasma catecholamine levels [10]–[12]. The increased BP and HR effects have been shown to be mediated mainly by sympathetic activation [6], [7], [12].

Despite frequent associations between NC and obesity, type 2 diabetes mellitus, and metabolic syndrome, few studies addressing cardiovascular consequences have been conducted in human NC [13]. BP and HR variation and day–night BP decrease (the so-called “dipping pattern”) are strongly linked to the sleep-wake circadian rhythm [14]. The nocturnal fall in BP has major clinical implications: a blunted nocturnal BP dip is associated with high risk of cardiovascular morbidity and mortality [15]–[19]. A meta-analysis showed that nighttime BP was a stronger predictor than daytime BP of adverse cardiovascular events [18]. Endothelial dysfunction is another predictive marker of cardiovascular morbidity and mortality [20]. Reactive hyperemia peripheral arterial tonometry (RH-PAT) is a non-invasive, operator-independent, easily reproducible technique that effectively predicts endothelial dysfunction (an early marker for atherosclerosis) and future adverse cardiac events [20]–[22].

Because hypocretin system is one of the factors involved in the complex interaction between sleep and the cardiovascular system, we tested the hypothesis that hypocretin deficiency may affect markers of cardiovascular risk. We measured nighttime and daytime ambulatory blood pressure (BP) and their night-to-day ratio, the digital pulse amplitude response (RH-PAT) to evaluate endothelial dysfunction in drug-free patients with narcolepsy-cataplexy (NC) compared to controls.

## Methods

### Subjects

All patients and controls gave their informed written consent to take part in the study which was approved by the Local Ethics Committee (University Hospital, Montpellier, France). Additional written consent was obtained from the parents of participants under the age of 18.

Fifty patients with typical NC (30 males, 20 females; aged 14 to 76 years; median age 34 years) were examined. NC was diagnosed according to the revised International Classification of Sleep Disorders [23], including the presence of EDS, clear-cut and frequent cataplexy, and HLA DQB1*0602 positivity. A lumbar puncture was performed in 17 patients to measure CSF hypocretin-1, and low levels (<110 pg/ml) [4] were systematically found. All participants underwent a standardized face-to-face clinical interview to determine age at NC onset, duration of disease, Epworth sleepiness score, and frequency of cataplexy. All patients were drug-free for NC at the time of evaluation: 33 were drug-naive and 17 were treated with psychostimulants (modafinil, n = 15; methylphenidate, n = 2), including 3 with anticataplectics (venlafaxine, n = 1; clomipramine, n = 2) and 2 with gamma-hydroxybutyrate. All treatments were suspended at least two weeks before the study.

Forty-two community-dwelling healthy control participants (16 males, 26 females; aged 20 to 53 years; median age 31.5 years) were recruited from local associative networks (Montpellier, France).

No patients or controls had any psychiatric disorder, based on DSM-IV TR criteria, and none was taking any medication known to influence sleep at the time of the study. Regarding the presence of medication influencing cardiovascular function, five subjects (four patients with NC and one control) were treated with an antihypertensive drug, two patients with a drug for diabetes, and one patient with a treatment for hyperlipidemia.

### Clinical and Biological Cardiovascular Risk Factors

All participants underwent a routine medical history and physical examination, and body-mass index (BMI) and waist and hip circumference were measured. All participants fasted for venous blood sampling between 7 and 8 AM, with similar procedures to assess for cardiovascular biological risk factors.

Presence of baseline cardiovascular risk factors was defined as follows: (1) hypertension as a conventional BP of at least 130 mm Hg systolic and 85 mm Hg diastolic, or as use of antihypertensive drugs; (2) diabetes mellitus as positive patient history, a fasting blood glucose concentration of at least 7.0 mmol/L, or use of antidiabetic drugs; (3) hyperlipidaemia as total serum cholesterol >240 mg/dL, triglyceride ≥150 mg/L, or treatment with lipid-lowering drugs; (4) metabolic syndrome diagnosed according to National Cholesterol Education Program Adult Treatment Panel III criteria [24]; (5) insulin resistance defined as the homeostasis model assessment (HOMA) [25], (6) personal history of coronary artery disease (CAD) or stroke; (7) history of CAD or stroke in first-degree relatives; (8) smoking history; and (9) alcohol intake.

### Polysomnographic Recordings

All patients (n = 50) and 23 controls underwent one night of polysomnography (PSG) recording in the sleep laboratory followed by the Multiple Sleep Latency Test (MSLT) the next day. Sleep recording included 3 EEG leads (C3, C4, and O2), 2 electroculograms, one chin electromyogram, and an electrocardiogram. Airflow was measured using a nasal cannula and mouth thermocouple, thoracoabdominal movement by respiratory belts (piezoelectric), and arterial oxygen saturation by finger pulse oximetry. Sleep recordings were manually scored by standard criteria for sleep stages as well as microarousals and respiratory events [26]. Obstructive sleep apneas were defined as complete cessation of airflow for >10 sec associated with thoracoabdominal movements, and hypopneas were defined as a ≥50% reduction in airflow plus a ≥3% drop in SpO2 and/or a microarousal. Indices of periodic leg movements (PLM) were calculated as PLMW (awake), PLMS (asleep), PLMS in REM and NREM sleep, and PLM-MAs (microarousal-associated) [27].

### Digital Pulse Amplitude

Digital pulse amplitude was measured between 7 and 8 AM after overnight fasting in all participants in supine position by RH-PAT using an Endo-PAT finger sensor (Itamar Medical Ltd, Caesarea, Israel) [21], [22]. Pulse amplitude was recorded electronically in both fingers and analyzed by a computerized automated algorithm to obtain average pulse amplitude for 30-second intervals from forearm cuff deflation up to 4 minutes. The RH-PAT index, a measure of endothelial dysfunction, was calculated as the natural logarithm of the average amplitude of the PAT signal after 90- to 120-second deflation divided by the average amplitude of the PAT signal during the 210 seconds prior to cuff inflation.

### 24-h Ambulatory Blood Pressure Monitoring

Twenty-four-hour ambulatory blood pressure monitoring (ABPM) was done on the non-dominant arm using a validated automatic oscillometer (i-MAPA®, Eutherapie) following the PSG-MSLT recording in 36 patients and in all controls (n = 42). All participants were instructed to continue their usual daily activities but without napping. BP and HR readings were automatically recorded at 15-minute intervals in daytime and 60-minute intervals at night. Daytime and nighttime were individually predetermined depending on the participants’ usual awake and sleep schedule. Recordings were analyzed to obtain average 24-hour daytime and nighttime systolic and diastolic BP and HR. Systolic and diastolic BP were used to assess dipping. A “non-dipping BP profile” was defined as a nocturnal BP dip<10% lower than daytime BP [19].

### Statistical Methods

Categorial variables for the sample are presented as percentages, and quantitative variables as medians with ranges. Distributions were mostly skewed, according to Shapiro-Wilk’s test. Chi square or Fisher’s exact tests were run to compare categorical variables between the two groups, and the Mann-Whitney test for continuous variables (table 1 and table 2). Spearman’s rank order correlations were applied to determine associations between two continuous variables. Univariate comparisons between patients with NC and controls were made using logistic regression analysis and quantified using odds ratios (OR) and 95% confidence intervals (CI) for exposure variables (pulse amplitude measures by RH-PAT and 24-hour ambulatory blood pressure). ORs evaluated the strength of the associations between exposure variables and the two groups (controls and patients). More precisely, OR is the risk of being narcoleptic when the subject is exposed. For the blood pressure example, OR means that for 1 variable unit (1 mmHg) increase, the risk of being narcoleptic is OR value. ORs were further adjusted for clinical and biological variables associated with narcolepsy (at p<0.10) in table 1 which could be potential confounders (excluding dislypidemia due to the low proportion of dyslipidemia in the sample and table’s cells have expected counts less than 5) using multivariate logistic regression models (table 3 and table 4). To identify factors associated with a low PAT ratio for the 90–120-second intervals, continuous variables were divided into two groups according to low or high PAT ratio using a 1.49 threshold (first tertile of the distribution). To obtain a sufficient statistical power, we decided to adjust the two groups for potential confounders instead of performing analysis on homogeneous sub-groups of patients or controls. The latter choice may be considered as particularly difficult as narcolepsy with cataplexy is an orphan disease associated with several comorbid conditions such as obesity. Similar procedures were applied for factors associated with a non-dipping vs. dipping BP profile, with significance at p<0.05. Statistical analyses were performed using SAS, version 9.2 (SAS Institute, Cary, NC, USA).

**Table 1:** Clinical and biological characteristics of patients with narcolepsy-cataplexy (n = 50) and controls (n = 42).

	*Controls N = 42*		*Narcoleptics N = 50*		
*Variable*	*n*	*%*	*n*	*%*	*p* (d)
Gender					
Male	16	38.10	30	60.00	0.04
Female	26	61.90	20	40.00	
Age (median [range])	31.50 [20.00–53.00]		34.00 [14.00–76.00]		0.73
Waist/hip ratio					
<1	35	100.00	43	91.49	0.13
≥1	0	0.00	4	8.51	
Body mass index (kg/m^2^)	21.40 [18.30–29.10]		25.11 [17.01–38.30]		<0.0001
Waist circumference, cm					
≤102 in men or ≤88 in women	31	86.11	37	78.72	0.39
>102 in men or >88 in women	5	13.89	10	21.28	
Smoker					
No	34	80.95	25	50.00	0.002
Yes	8	19.05	25	50.00	
Alcohol intake					
No	21	52.50	26	53.06	0.62
Occasional	15	37.50	15	30.61	
Frequent(a)	4	10.00	8	16.33	
Family history of CAD(b)					
No	37	92.50	40	86.96	0.49
Yes	3	7.50	6	13.04	
Diabetes mellitus					
No	42	100.00	48	96.00	0.50
Yes	0	0.00	2	4.00	
Hypertension					
No	41	97.62	46	92.00	0.37
Yes	1	2.38	4	8.00	
Dyslipidemia					
No	42	100.00	44	88.00	0.03
Yes	0	0.00	6	12.00	
Metabolic syndrome					
No	39	100.00	43	91.49	0.12
Yes	0	0.00	4	8.51	
HOMA-IR(c)					
≤2.5	37	94.87	44	97.78	0.59
>2.5	2	5.13	1	2.22	
C-reactive protein (mg/L)					
≤0.5	14	37.84	14	31.11	0.43
[0.5–2.0]	14	37.84	14	31.11	
>2.0	9	24.32	17	37.78	

(a) Frequent intake was defined as at least daily ingestion;

(b) CAD: coronary artery disease.

(c) HOMA-IR: homeostasis model assessment (HOMA), IR: insulin resistance.

(d) p-values from Chi-square test or Fisher’s exact test or Mann-Whitney test.

**Table 2:** Polysomnography and multiple sleep latency tests in patients with narcolepsy-cataplexy (n = 50) and in controls (n = 23).

	*Controls N = 23*	*Narcoleptics N = 50*	*p* (e)
	Median [Range]	Median [Range]	
Total sleep time	417.00 [289.00–474.00]	418.50 [275.00–539.00]	0.37
Sleep efficiency	87.35 [58.93–95.39]	86.05 [57.69–96.52]	0.29
N1(%)	3.49 [0.47–14.65]	9.25 [1.67–38.40]	<0.001
N2 (%)	55.40 [29.66–68.67]	47.60 [17.86–60.91]	0.001
N3 (%)	20.96 [7.20–44.56]	18.81 [7.51–39.49]	0.02
REM sleep (%)	16.60 [9.44–27.40]	23.71 [10.80–37.00]	<0.001
REM sleep latency	85.00 [61.00–206.00]	9.50 [0.00–274.00]	<0.001
Sleep latency	15.00 [2.00–84.00]	4.00 [0.00–18.00]	<0.001
WASO(a)	37.00 [11.00–120.00]	68.00 [15.00–189.00]	0.003
AHI(b)	1.50 [0.00–9.20]	4.10 [0.00–44.21]	0.02
PLMW index(c)	2.96 [0.00–102.22]	5.68 [0.00–48.57]	0.24
PLMS index	0.00 [0.00–7.91]	2.10 [0.00–103.84]	0.003
PLMS-MA	0.00 [0.00–1.24]	0.29 [0.00–8.23]	0.01
Microarousal index	12.31 [4.19–27.10]	14.10 [0.00–47.96]	0.42
Mean SaO2	96.00 [88.00–98.00]	96.00 [92.00–98.00]	0.85
SaO2 less than 90% duration	0.02 [0.00–46.57]	0.01 [0.00–6.41]	0.51
O2 desaturation index	2.16 [0.00–36.33]	3.20 [0.00–51.56]	0.63
Mean sleep latency, min	17.31 [8.60–20.00]	5.40 [0.40–16.80]	<0.001
SOREMPs(d), n	0.00 [0.00–1.00]	4.00 [0.00–5.00]	<0.001

Data are expressed as medians [Min-Max].

(a) WASO: Wake time after sleep onset;

(b) AHI: Apnea hypopnea index;

(c) PLM: periodic leg movements (PLMS: in sleep; PLMW: in wake time; PLMS-MA: with microarousals);

(d) SOREMPs: Sleep Onset REM Periods;

(e) p-values from Mann-Whitney test.

**Table 3:** Pulse amplitude measures by reactive hyperemia with finger plethysmography (reactive hyperemia peripheral arterial tonometry – RH-PAT) in patients with narcolepsy-cataplexy (n = 50) compared to controls (n = 42).

	*Controls*	*Narcoleptics*				
	*N = 42*	*N = 50*	*OR [95% CI]*	*p*	*OR [95% CI]* (b)	*P* (b)
RH-PAT(a) 90–120 measures						
Median [range]	1.83 [0.82–3.52]	1.73 [0.88–3.70]	0.76 [0.43–1.33](c)	0.33	0.78 [0.41–1.51](c)	0.46
Log RH- PAT 90–120 measures	0.65 (0.39)	0.58 (0.36)	0.62 [0.20–1.87](c)	0.39	0.71 [0.20–2.58](c)	0.60
Mean (SD)	0.65 (0.39)	0.58 (0.36)	0.62 [0.20–1.87](c)	0.39	0.71 [0.20–2.58](c)	0.60
Log RH-PAT 90–120 measures						
<0.4 (n, %)	13 30.95	19 38.00	1	0.48	1	0.76
≥0.4 (n, %)	29 69.05	31 62.00	0.73 [0.31–1.74]		0.85 [0.31–2.35]	
RH-PAT 90–120 measures						
≤1.49 (n, %)	13 30.95	17 34.00	1	0.71	1	0.88
[1.49–2.22] (n, %)	13 30.95	18 36.00	1.06 [0.38–2.92]		1.05 [0.33–3.38]	
>2.22 (n, %)	16 38.10	15 30.00	0.72 [0.26–1.97]		0.79 [0.24–2.57]	

(a) RH-PAT: reactive hyperemia peripheral arterial tonometry.

(b) Adjusted for gender, smoking, BMI.

(c) OR for 1-unit increase; OR means that for 1 variable unit increase, the risk of being narcoleptic was OR value.

**Table 4:** 24-hour ambulatory blood pressure monitoring of patients with narcolepsy-cataplexy (n = 36) and of controls (n = 42).

	*Controls N = 42*	*Narcoleptics N = 36*	*OR [95% CI]* (b)	*p*	*OR [95% CI]^(b,c)^*	*p* (c)
	*Median [Range]*	*Median [Range]*				
Ambulatory blood pressure (mmHg)						
24-h systolic	112.00 [98.00–132.00]	115.50 [97.00–165.00]	1.03 [0.99–1.09]	0.17	0.99 [0.93;1.05]	0.73
24-h diastolic	72.50 [61.00–85.00]	70.50 [49.00–113.00]	0.99 [0.94–1.05]	0.72	0.94 [0.88;1.01]	0.07
Daytime systolic	116.50 [101.00–139.00]	120.50 [99.00–168.00]	1.03 [0.98–1.07]	0.23	0.98 [0.93;1.04]	0.51
Daytime diastolic	76.00 [66.00–87.00]	73.00 [52.00–115.00]	1.03 [0.98–1.07]	0.23	0.98 [0.93;1.04]	0.51
Mean daytime pressure(a)	90.00 [79.33–100.33]	90.00 [69.33–132.67]	0.99 [0.94–1.04]	0.69	0.93 [0.87;0.99]	0.03
Daytime heart rate (/min)	77.00 [57.00–97.00]	70.00 [56.00–89.00]	1.00 [0.95–1.06]	0.93	0.94 [0.88;1.00]	0.06
Nighttime systolic	102.00 [69.00–122.00]	107.00 [93.00–155.00]	1.05 [1.00–1.10]	0.05	1.02 [0.97;1.08]	0.40
Nighttime diastolic	61.00 [47.00–76.00]	63.50 [46.00–109.00]	1.05 [0.98–1.11]	0.15	1.01 [0.94;1.08]	0.88
Mean nighttime pressure	75.00 [62.33–90.00]	77.67 [63.00–124.33]	1.05 [0.99–1.12]	0.10	1.01 [0.94;1.08]	0.77
Nighttime heart rate (/min)	61.50 [50.00–83.00]	61.00 [46.00–79.00]	0.97 [0.92–1.03]	0.35	0.99 [0.93;1.05]	0.70
Systolic dip	12.36 [–6.00–22.10]	10.63 [−2.10–23.13]	0.94 [0.87–1.02]	0.15	0.92 [0.84;1.01]	0.08
Diastolic dip	17.60 [−5.40–31.70]	13.15 [−1.80–26.40]	0.90 [0.83–0.97]	0.006	0.87 [0.79;0.96]	0.006
Mean pressure dip	13.67 [−6.00–29.00]	11.67 [−1.00–23.00]	0.90 [0.83–0.98]	0.02	0.86 [0.77;0.96]	0.01

(a) (PAS-2*PAD)/3).

(b) OR for 1-unit increase; OR means that for 1 variable unit (1 mmHg) increase, the risk of being narcoleptic was OR value.

(c) Adjusted for gender, smoking, BMI.

## Results

### Clinical and Biological Characteristics

Table 1 presents the clinical and biological characteristics of patients and controls, revealing a larger proportion of men with NC. More patients than controls were currently smokers (50.0% vs. 19.1%, p = 0.002), obese (27.6% vs. 0%, p<0.0001) and with dyslipidemia (12% vs 0, p = 0.03). Other clinical and biological variables did not differ significantly between groups (Table 1). Based on case and control differences for gender, smoking, and BMI, all between-group results were further adjusted accordingly. Two patients with NC had a personal history of CAD, with none for controls.

### Sleep Parameters

As expected, Table 2 depicts that patients with NC had significantly shorter sleep and REM sleep latency, longer wake time after sleep onset, greater percentages of N1 and REM sleep, and lower N2 and N3 sleep. Between-group comparison showed a higher AHI index, and PLMS index with and without micro-arousals in NC, with no significant difference for PLMW, mean nocturnal O2 saturation, or desaturation index. Only 6.4% of patients with NC had AHI >30 per hour, without any in the controls. MSLT results revealed shorter mean sleep latency in NC with a higher number of sleep onset REM periods (SOREMPS) (Table 2). No controls had abnormal mean sleep latency (<8 minutes), and only one control had one SOREMP.

### Endothelial Function

Regardless of the distribution (continuous or categorical), RH-PAT 90–120 measurements were not significantly lower in the NC group than in controls, before and after adjusting for gender, smoking, and BMI (OR = 0.76 95% CI = 0.43–1.33, OR = 0.78 95% CI = 0.41–1.51, respectively) (Table 3). RH-PAT of the whole population divided into tertiles revealed a 1.49 cut-off for the first tertile, without any difference between the percentage of patients and controls below this threshold. Comparing patients with NC with a low amplitude response to those with a larger response (Log RH-PAT 90–120 s <0.4, n = 19 vs >0.4, n = 31), we failed to report any difference with gender, age, BMI, clinical and biological data, and sleep variables (Table 3). As previous psychostimulant intake may have affected the endothelial function, additional analyses were performed but the results remained similar between drug-naive, drug-free patients for at least 15 days, and controls.

### 24-hour Ambulatory Systolic and Diastolic BP

**Figure 1:**
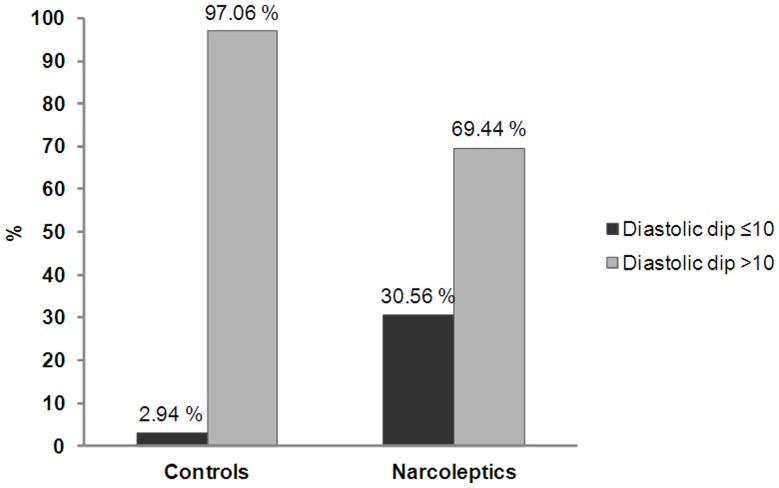
Non-dipping diastolic BP profile in patients with narcolepsy-cataplexy (n = 36) and in controls (n = 42). Diastolic non-dippers (defined as a nocturnal diastolic BP dip<10% lower than daytime BP) were significantly higher in patients with narcolepsy-cataplexy compared to controls (30.56% vs 2.94%, p = 0.002).

Patients with NC showed lower daytime diastolic BP and HR than controls, with similar tendency for the mean daytime pressure (Table 4). Large between-group differences were noted for diastolic and mean pressure dips (i.e. >10% drop in BP during sleep): lower in NC, with a similar tendency for systolic dips. Figure 1 shows a higher percentage of diastolic non-dippers in patients with NC compared to controls (30.56% vs 2.94%, p = 0.002). When the non-dipping night-to-day ratio was below 15% (instead of 10%), results also showed a higher percentage of diastolic non-dippers in NC compared to controls (63.89% vs. 32.35%, p = 0.0086, OR = 5.12 95% CI [1.51–17.30] after adjusting for gender, smoking, and BMI). We further analyzed factors associated with the diastolic dip (<10% drop during sleep) in patients with NC. Hence, the diastolic non-dipper status was associated with dyslipidemia (22.3% in non-dipper vs 0% in dipper, p = 0.02), with a similar tendency for REM sleep percentage (27.1% vs 22.2%, p = 0.08). No other between-dipper group differences were found for gender, age, disease duration, BMI, clinical and biological data, and other sleep variables (including sleep efficiency, total sleep time, sleep stage %, mean PLMS and AHI indexes, and number of O2 desaturations). As previous psychostimulant intake may have affected BP, additional analyses were performed but results remained unchanged between drug-naive, drug-free patients for at least 15 days, and controls.

We noted in the whole population a negative correlation between mean diastolic dip and REM sleep percentage (r = −0.46, p<0.0003) and number of SOREMPs (r = −0.44, P = 0.001), and a positive correlation with mean sleep latency on the MSLT (r = 0.49, P = 0.0002). No association was found between RH-PAT, and mean 24-h blood pressure or dipper status.

## Discussion

For the first time, 24-hour ambulatory blood pressure (BP) monitoring and the peripheral endothelial function were reported in a large group of drug-free NC patients in comparison to controls. Although the digital pulse amplitude response, measured by reactive hyperemia-peripheral artery tonometry (RH-PAT), was substantially similar between NC and controls, one-third of NC patients were non-dippers vs. only 3% of controls. Non-dipper diastolic BP status was strongly associated with NC by an up to 12-fold higher odds ratio, with significant associations with nocturnal and diurnal REM sleep, and objective daytime sleepiness.

Patients with NC showed lower daytime diastolic BP, and HR than controls. This could be partially explained by lesser physical activity in patients due to severe daytime hypersomnia [28]. The literature reports that mean sleeping BP and the 24-h BP pattern (dipping vs. non-dipping) are more reliable indicators than daytime BP, and better predictors of cardiac morbidity and mortality risks [15]–[19]. We found a nighttime non-dipping diastolic BP pattern in NC, with 31% of patients failing to show the 10% physiological sleep-related decline, and 64% failing to show the 15% fall point. Although systolic dip was also lower in NC compared to controls, no between-group association was found for non-systolic dipper status. Our ambulatory BP monitoring had some limitations, performing every 15 minutes during the day but every 60 minutes at night to preserve sleep continuity but precluding large nighttime data. During the ambulatory monitoring, all participants were instructed to continue their usual daily activities but without napping; however we do not have objective information on the sleep/wake pattern during BP recording.

We recently reported lower cardiac activation associated with periodic leg movements during sleep (PLMS) in NC, probably due to changes in baroreflex sensitivity [29]. Both lower PLMS-related HR response and the absence of or reduction in the nocturnal dip may be considered highly sensitive predictors of cardiovascular morbidity and mortality [15]–[19], [30]–[31]. However, based on discrepancies between the general non-dipper profile and the one of NC with low daytime diastolic BP and HR without any increased of hypertension, we may also further hypothesize the absence of higher cardiovascular risk in NC.

One strength of this study is the between-group comparison of robust cardiovascular markers in drug-free patients with NC by considering during meaning time potential clinical and biological confounding factors. Patients with NC were more smokers, obese and with dyslipidemia than controls but without any difference for hypertension, diabetes mellitus, metabolic syndrome, insulin resistance, and personal history of coronary artery disease or stroke. We found associations between diastolic non-dipper status and dyslipidemia in NC with a similar tendency for REM sleep percentage [15], [16], [19]. Importantly, we found negative correlations between mean diastolic dip, REM sleep percentage, and number of SOREMPs, and a positive correlation with mean daytime sleep latency. Our findings suggest that the abnormal night-to-day cardiovascular regulation found in NC is sleep related, being impacted by both REM sleep and hypersomnia, hallmarks of narcolepsy-cataplexy. Unlike NREM sleep, REM sleep has been associated with marked sympathetic activation and with BP and HR instability [32]. Moreover, vasoconstriction in active skeletal muscles during REM sleep, depending on sympathetic outflow, is required to prevent drops in systemic arterial pressure [33]. A blunted sleep-related decrease in BP was reported in animal models of narcolepsy leading to prominent sleep-related increases in BP during REM sleep [9]. Recent data also suggest that patients with NC displayed a nighttime nondipping BP pattern with increased of systolic BP during nighttime REM sleep [34]. Altogether these results suggest a decreased night-to-day BP pattern in human NC, attributable to a potentially clinically significant hypocretin deficiency.

A reliable, non-invasive method using a PAT sensor was developed to detect vascular changes in patients at high cardiovascular risk [20]–[22]. Recent population-based studies further confirmed that the PAT ratio independently predicts cardiovascular events, and early detection facilitates therapy and prognosis [20]–[22]. In our study, the endothelium-dependent vasodilatation response was similar between NC and controls, suggesting normal arterial smooth muscle cell functioning. We failed to report any determinant of endothelial function results in NC. These results may contradict previous studies on PAT in sleep disorders especially in sleep apnea syndrome [35], however only few patients (6.4%) with NC had AHI above 30, allowing for potential type II errors. Overall, our RH-PAT results suggest a preserved vasomotor microvessel function in NC that may be related to low daytime BP findings.

Unlike a recent study [36], we found no association between RH-PAT results, mean 24-h blood pressure, and dipper status in either patients or controls, possibly because most participants had normal diurnal BP at baseline (94.6%). Literature reports that significant percentage of patients with NC are obese, exhibit type II diabetes or metabolic syndrome [1], [13], and are taking life-long treatments with psychostimulants, known to affect the autonomic nervous and cardiovascular systems [1]. Altogether these results may indicate an increased risk for cardiovascular events in NC but the low daytime diastolic BP and HR without any associated hypertension found in the present study may also attenuate this potential risk. Given the lack of relevant data regarding cardiovascular co-morbidities and mortality in the literature, long-term prospective observational cohorts should be examined to assess these issues including measurements of endothelial dysfunction and 24-hour-BP.

In conclusion, we demonstrated a large percentage of diastolic non-dippers in patients with NC compared to controls, with associations with REM sleep regulation. The blunted sleep-related BP dip in NC may be of clinical relevance as it may indicate increased risk for cardiovascular events.
